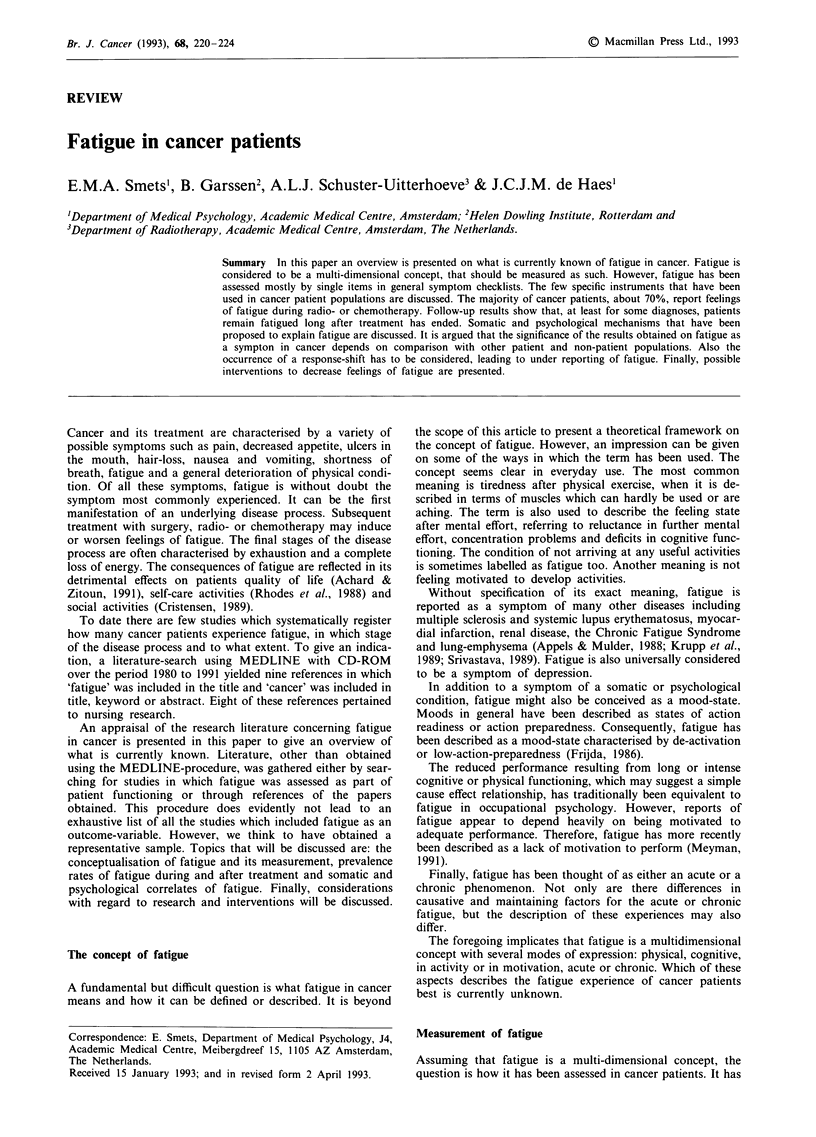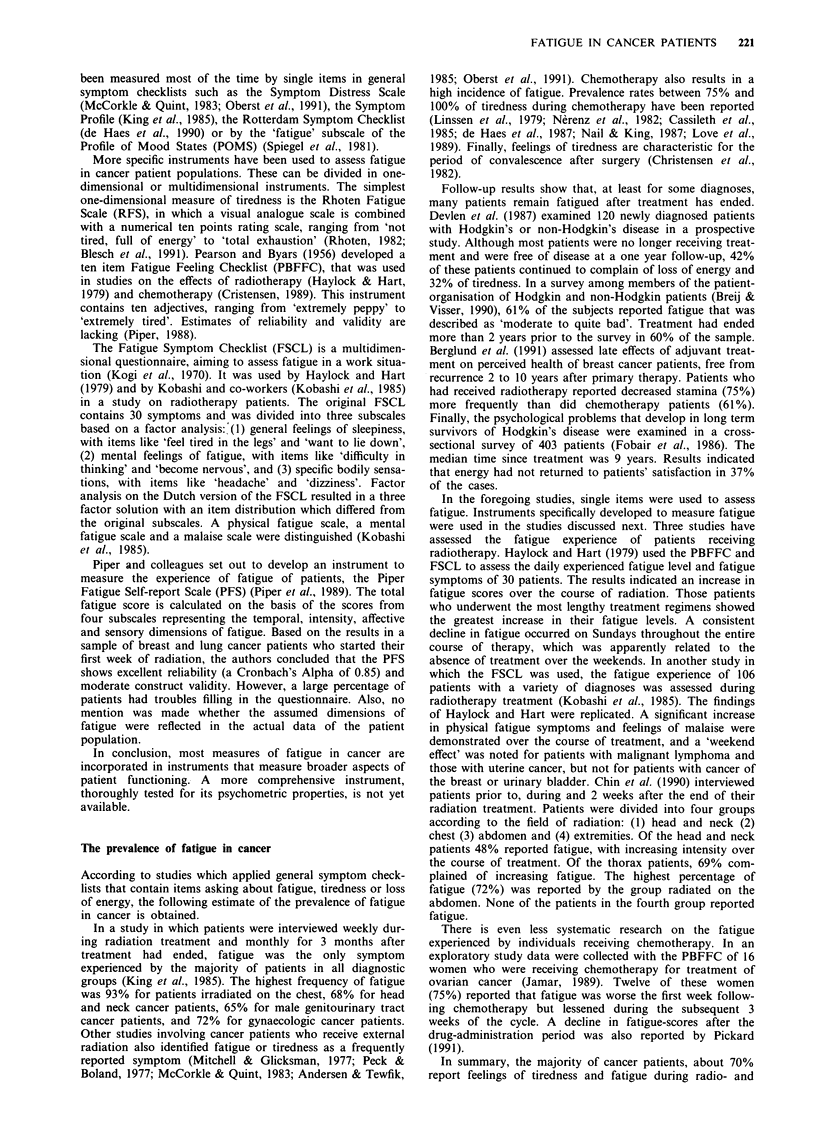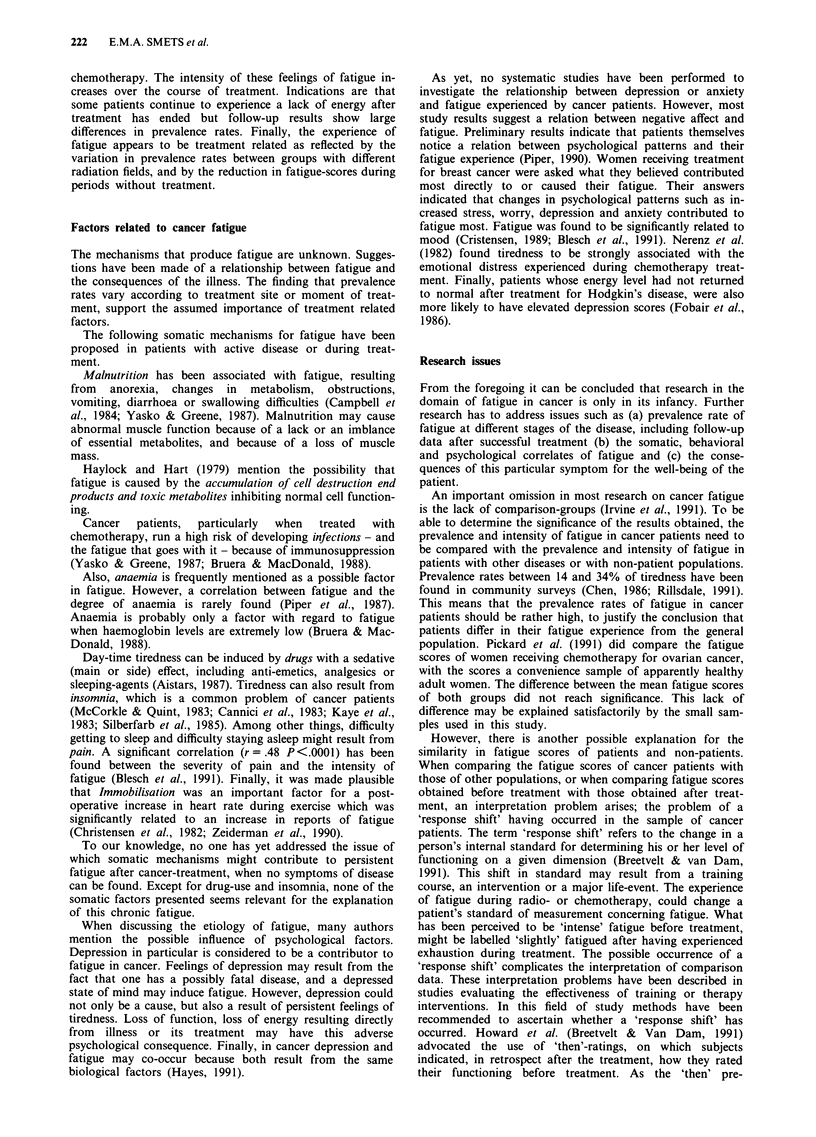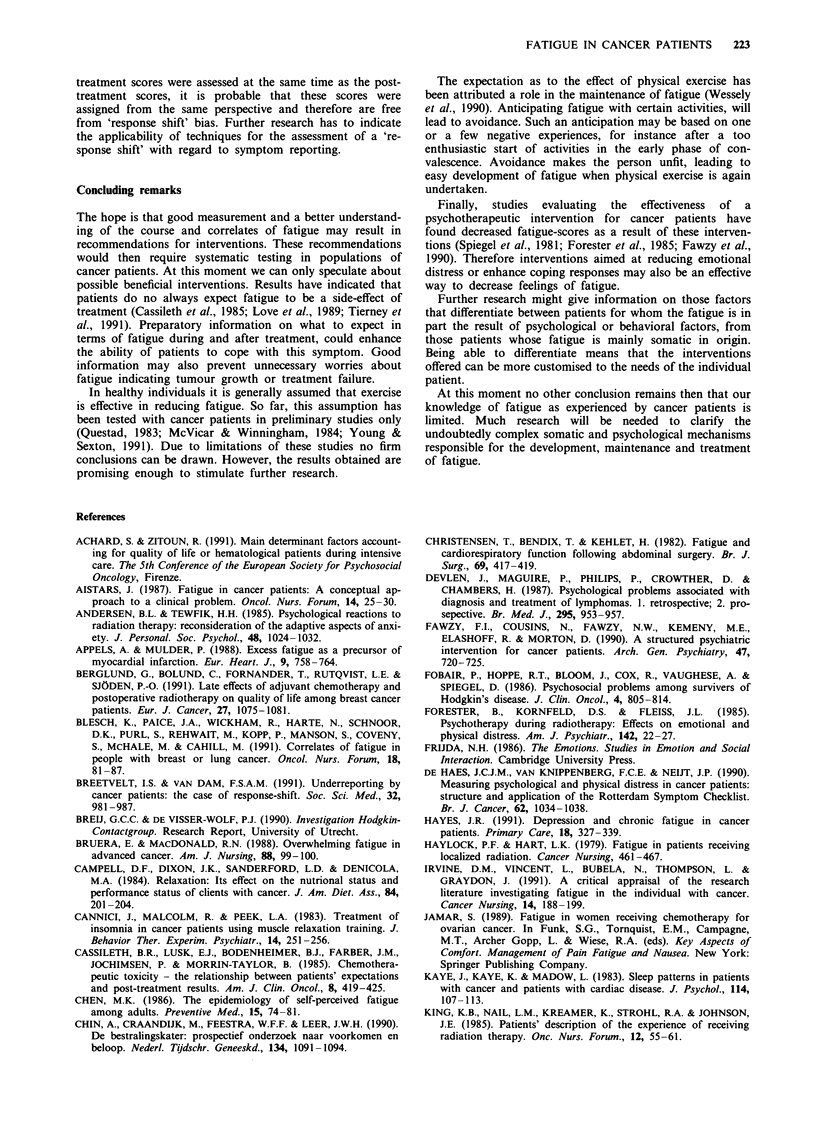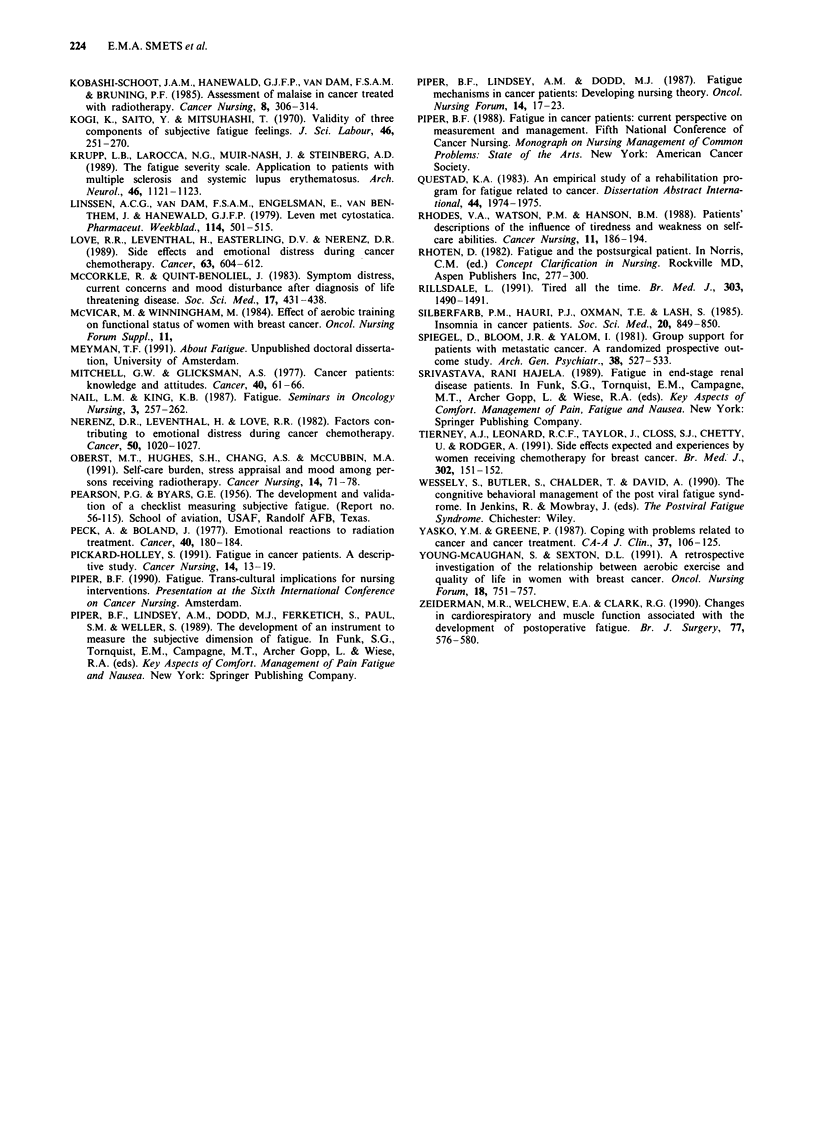# Fatigue in cancer patients.

**DOI:** 10.1038/bjc.1993.319

**Published:** 1993-08

**Authors:** E. M. Smets, B. Garssen, A. L. Schuster-Uitterhoeve, J. C. de Haes

**Affiliations:** Department of Medical Psychology, Academic Medical Centre, Amsterdam, The Netherlands.

## Abstract

In this paper an overview is presented on what is currently known of fatigue in cancer. Fatigue is considered to be a multi-dimensional concept, that should be measured as such. However, fatigue has been assessed mostly by single items in general symptom checklists. The few specific instruments that have been used in cancer patient populations are discussed. The majority of cancer patients, about 70%, report feelings of fatigue during radio- or chemotherapy. Follow-up results show that, at least for some diagnoses, patients remain fatigued long after treatment has ended. Somatic and psychological mechanisms that have been proposed to explain fatigue are discussed. It is argued that the significance of the results obtained on fatigue as a symptom in cancer depends on comparison with other patient and non-patient populations. Also the occurrence of a response-shift has to be considered, leading to under reporting of fatigue. Finally, possible interventions to decrease feelings of fatigue are presented.


Br. J. Cancer (1993), **68**, 220–224 © Macmillan Press Ltd., 1993

**REVIEW**

### Fatigue in cancer patients

E.M.A. Smets[1], B. Garssen[2], A.L.J. Schuster-Uitterhoeve[3] & J.C.J.M. de Haes[1]

*[1]Department of Medical Psychology, Academic Medical Centre, Amsterdam; [2]Helen Dowling Institute, Rotterdam and [3]Department of Radiotherapy, Academic Medical Centre, Amsterdam, The Netherlands.*

**Summary** In this paper an overview is presented on what is currently known of fatigue in cancer. Fatigue is considered to be a multi-dimensional concept, that should be measured as such. However, fatigue has been assessed mostly by single items in general symptom checklists. The few specific instruments that have been used in cancer patient populations are discussed. The majority of cancer patients, about 70%, report feelings of fatigue during radio- or chemotherapy. Follow-up results show that, at least for some diagnoses, patients remain fatigued long after treatment has ended. Somatic and psychological mechanisms that have been proposed to explain fatigue are discussed. It is argued that the significance of the results obtained on fatigue as a sympton in cancer depends on comparison with other patient and non-patient populations. Also the occurrence of a response-shift has to be considered, leading to under reporting of fatigue. Finally, possible interventions to decrease feelings of fatigue are presented.

Cancer and its treatment are characterised by a variety of possible symptoms such as pain, decreased appetite, ulcers in the mouth, hair-loss, nausea and vomiting, shortness of breath, fatigue and a general deterioration of physical condition. Of all these symptoms, fatigue is without doubt the symptom most commonly experienced. It can be the first manifestation of an underlying disease process. Subsequent treatment with surgery, radio- or chemotherapy may induce or worsen feelings of fatigue. The final stages of the disease process are often characterised by exhaustion and a complete loss of energy. The consequences of fatigue are reflected in its detrimental effects on patients quality of life (Achard & Zitoun, 1991), self-care activities (Rhodes *et al.*, 1988) and social activities (Cristensen, 1989).

To date there are few studies which systematically register how many cancer patients experience fatigue, in which stage of the disease process and to what extent. To give an indication, a literature-search using MEDLINE with CD-ROM over the period 1980 to 1991 yielded nine references in which 'fatigue' was included in the title and 'cancer' was included in title, keyword or abstract. Eight of these references pertained to nursing research.

An appraisal of the research literature concerning fatigue in cancer is presented in this paper to give an overview of what is currently known. Literature, other than obtained using the MEDLINE-procedure, was gathered either by searching for studies in which fatigue was assessed as part of patient functioning or through references of the papers obtained. This procedure does evidently not lead to an exhaustive list of all the studies which included fatigue as an outcome-variable. However, we think to have obtained a representative sample. Topics that will be discussed are: the conceptualisation of fatigue and its measurement, prevalence rates of fatigue during and after treatment and somatic and psychological correlates of fatigue. Finally, considerations with regard to research and interventions will be discussed.

### The concept of fatigue

A fundamental but difficult question is what fatigue in cancer means and how it can be defined or described. It is beyond the scope of this article to present a theoretical framework on the concept of fatigue. However, an impression can be given on some of the ways in which the term has been used. The concept seems clear in everyday use. The most common meaning is tiredness after physical exercise, when it is described in terms of muscles which can hardly be used or are aching. The term is also used to describe the feeling state after mental effort, referring to reluctance in further mental effort, concentration problems and deficits in cognitive functioning. The condition of not arriving at any useful activities is sometimes labelled as fatigue too. Another meaning is not feeling motivated to develop activities.

Without specification of its exact meaning, fatigue is reported as a symptom of many other diseases including multiple sclerosis and systemic lupus erythematosus, myocardial infarction, renal disease, the Chronic Fatigue Syndrome and lung-emphysema (Appels & Mulder, 1988; Krupp *et al.*, 1989; Srivastava, 1989). Fatigue is also universally considered to be a symptom of depression.

In addition to a symptom of a somatic or psychological condition, fatigue might also be conceived as a mood-state. Moods in general have been described as states of action readiness or action preparedness. Consequently, fatigue has been described as a mood-state characterised by de-activation or low-action-preparedness (Frijda, 1986).

The reduced performance resulting from long or intense cognitive or physical functioning, which may suggest a simple cause effect relationship, has traditionally been equivalent to fatigue in occupational psychology. However, reports of fatigue appear to depend heavily on being motivated to adequate performance. Therefore, fatigue has more recently been described as a lack of motivation to perform (Meyman, 1991).

Finally, fatigue has been thought of as either an acute or a chronic phenomenon. Not only are there differences in causative and maintaining factors for the acute or chronic fatigue, but the description of these experiences may also differ.

The foregoing implicates that fatigue is a multidimensional concept with several modes of expression: physical, cognitive, in activity or in motivation, acute or chronic. Which of these aspects describes the fatigue experience of cancer patients best is currently unknown.

### Measurement of fatigue

Assuming that fatigue is a multi-dimensional concept, the question is how it has been assessed in cancer patients. It has

Correspondence: E. Smets, Department of Medical Psychology, J4, Academic Medical Centre, Meibergdreef 15, 1105 AZ Amsterdam, The Netherlands.
Received 15 January 1993; and in revised form 2 April 1993.

been measured most of the time by single items in general symptom checklists such as the Symptom Distress Scale (McCorkle & Quint, 1983; Oberst *et al.*, 1991), the Symptom Profile (King *et al.*, 1985), the Rotterdam Symptom Checklist (de Haes *et al.*, 1990) or by the 'fatigue' subscale of the Profile of Mood States (POMS) (Spiegel *et al.*, 1981).

More specific instruments have been used to assess fatigue in cancer patient populations. These can be divided in one-dimensional or multidimensional instruments. The simplest one-dimensional measure of tiredness is the Rhoten Fatigue Scale (RFS), in which a visual analogue scale is combined with a numerical ten points rating scale, ranging from 'not tired, full of energy' to 'total exhaustion' (Rhoten, 1982; Blesch *et al.*, 1991). Pearson and Byars (1956) developed a ten item Fatigue Feeling Checklist (PBFFC), that was used in studies on the effects of radiotherapy (Haylock & Hart, 1979) and chemotherapy (Cristensen, 1989). This instrument contains ten adjectives, ranging from 'extremely peppy' to 'extremely tired'. Estimates of reliability and validity are lacking (Piper, 1988).

The Fatigue Symptom Checklist (FSCL) is a multidimensional questionnaire, aiming to assess fatigue in a work situation (Kogi *et al.*, 1970). It was used by Haylock and Hart (1979) and by Kobashi and co-workers (Kobashi *et al.*, 1985) in a study on radiotherapy patients. The original FSCL contains 30 symptoms and was divided into three subscales based on a factor analysis: (1) general feelings of sleepiness, with items like 'feel tired in the legs' and 'want to lie down', (2) mental feelings of fatigue, with items like 'difficulty in thinking' and 'become nervous', and (3) specific bodily sensations, with items like 'headache' and 'dizziness'. Factor analysis on the Dutch version of the FSCL resulted in a three factor solution with an item distribution which differed from the original subscales. A physical fatigue scale, a mental fatigue scale and a malaise scale were distinguished (Kobashi *et al.*, 1985).

Piper and colleagues set out to develop an instrument to measure the experience of fatigue of patients, the Piper Fatigue Self-report Scale (PFS) (Piper *et al.*, 1989). The total fatigue score is calculated on the basis of the scores from four subscales representing the temporal, intensity, affective and sensory dimensions of fatigue. Based on the results in a sample of breast and lung cancer patients who started their first week of radiation, the authors concluded that the PFS shows excellent reliability (a Cronbach's Alpha of 0.85) and moderate construct validity. However, a large percentage of patients had troubles filling in the questionnaire. Also, no mention was made whether the assumed dimensions of fatigue were reflected in the actual data of the patient population.

In conclusion, most measures of fatigue in cancer are incorporated in instruments that measure broader aspects of patient functioning. A more comprehensive instrument, thoroughly tested for its psychometric properties, is not yet available.

### The prevalence of fatigue in cancer

According to studies which applied general symptom checklists that contain items asking about fatigue, tiredness or loss of energy, the following estimate of the prevalence of fatigue in cancer is obtained.

In a study in which patients were interviewed weekly during radiation treatment and monthly for 3 months after treatment had ended, fatigue was the only symptom experienced by the majority of patients in all diagnostic groups (King *et al.*, 1985). The highest frequency of fatigue was 93% for patients irradiated on the chest, 68% for head and neck cancer patients, 65% for male genitourinary tract cancer patients, and 72% for gynaecologic cancer patients. Other studies involving cancer patients who receive external radiation also identified fatigue or tiredness as a frequently reported symptom (Mitchell & Glicksman, 1977; Peck & Boland, 1977; McCorkle & Quint, 1983; Andersen & Tewfik, 1985; Oberst *et al.*, 1991). Chemotherapy also results in a high incidence of fatigue. Prevalence rates between 75% and 100% of tiredness during chemotherapy have been reported (Linssen *et al.*, 1979; Nèrenz *et al.*, 1982; Cassileth *et al.*, 1985; de Haes *et al.*, 1987; Nail & King, 1987; Love *et al.*, 1989). Finally, feelings of tiredness are characteristic for the period of convalescence after surgery (Christensen *et al.*, 1982).

Follow-up results show that, at least for some diagnoses, many patients remain fatigued after treatment has ended. Devlen *et al.* (1987) examined 120 newly diagnosed patients with Hodgkin's or non-Hodgkin's disease in a prospective study. Although most patients were no longer receiving treatment and were free of disease at a one year follow-up, 42% of these patients continued to complain of loss of energy and 32% of tiredness. In a survey among members of the patient-organisation of Hodgkin and non-Hodgkin patients (Breij & Visser, 1990), 61% of the subjects reported fatigue that was described as 'moderate to quite bad'. Treatment had ended more than 2 years prior to the survey in 60% of the sample. Berglund *et al.* (1991) assessed late effects of adjuvant treatment on perceived health of breast cancer patients, free from recurrence 2 to 10 years after primary therapy. Patients who had received radiotherapy reported decreased stamina (75%) more frequently than did chemotherapy patients (61%). Finally, the psychological problems that develop in long term survivors of Hodgkin's disease were examined in a cross-sectional survey of 403 patients (Fobair *et al.*, 1986). The median time since treatment was 9 years. Results indicated that energy had not returned to patients' satisfaction in 37% of the cases.

In the foregoing studies, single items were used to assess fatigue. Instruments specifically developed to measure fatigue were used in the studies discussed next. Three studies have assessed the fatigue experience of patients receiving radiotherapy. Haylock and Hart (1979) used the PBFFC and FSCL to assess the daily experienced fatigue level and fatigue symptoms of 30 patients. The results indicated an increase in fatigue scores over the course of radiation. Those patients who underwent the most lengthy treatment regimens showed the greatest increase in their fatigue levels. A consistent decline in fatigue occurred on Sundays throughout the entire course of therapy, which was apparently related to the absence of treatment over the weekends. In another study in which the FSCL was used, the fatigue experience of 106 patients with a variety of diagnoses was assessed during radiotherapy treatment (Kobashi *et al.*, 1985). The findings of Haylock and Hart were replicated. A significant increase in physical fatigue symptoms and feelings of malaise were demonstrated over the course of treatment, and a 'weekend effect' was noted for patients with malignant lymphoma and those with uterine cancer, but not for patients with cancer of the breast or urinary bladder. Chin *et al.* (1990) interviewed patients prior to, during and 2 weeks after the end of their radiation treatment. Patients were divided into four groups according to the field of radiation: (1) head and neck (2) chest (3) abdomen and (4) extremities. Of the head and neck patients 48% reported fatigue, with increasing intensity over the course of treatment. Of the thorax patients, 69% complained of increasing fatigue. The highest percentage of fatigue (72%) was reported by the group radiated on the abdomen. None of the patients in the fourth group reported fatigue.

There is even less systematic research on the fatigue experienced by individuals receiving chemotherapy. In an exploratory study data were collected with the PBFFC of 16 women who were receiving chemotherapy for treatment of ovarian cancer (Jamar, 1989). Twelve of these women (75%) reported that fatigue was worse the first week following chemotherapy but lessened during the subsequent 3 weeks of the cycle. A decline in fatigue-scores after the drug-administration period was also reported by Pickard (1991).

In summary, the majority of cancer patients, about 70% report feelings of tiredness and fatigue during radio- and chemotherapy. The intensity of these feelings of fatigue increases over the course of treatment. Indications are that some patients continue to experience a lack of energy after treatment has ended but follow-up results show large differences in prevalence rates. Finally, the experience of fatigue appears to be treatment related as reflected by the variation in prevalence rates between groups with different radiation fields, and by the reduction in fatigue-scores during periods without treatment.

### Factors related to cancer fatigue

The mechanisms that produce fatigue are unknown. Suggestions have been made of a relationship between fatigue and the consequences of the illness. The finding that prevalence rates vary according to treatment site or moment of treatment, support the assumed importance of treatment related factors.

The following somatic mechanisms for fatigue have been proposed in patients with active disease or during treatment.

*Malnutrition* has been associated with fatigue, resulting from anorexia, changes in metabolism, obstructions, vomiting, diarrhoea or swallowing difficulties (Campbell *et al.*, 1984; Yasko & Greene, 1987). Malnutrition may cause abnormal muscle function because of a lack or an imblance of essential metabolites, and because of a loss of muscle mass.

Haylock and Hart (1979) mention the possibility that fatigue is caused by the *accumulation of cell destruction end products and toxic metabolites* inhibiting normal cell functioning.

Cancer patients, particularly when treated with chemotherapy, run a high risk of developing *infections* – and the fatigue that goes with it – because of immunosuppression (Yasko & Greene, 1987; Bruera & MacDonald, 1988).

Also, *anaemia* is frequently mentioned as a possible factor in fatigue. However, a correlation between fatigue and the degree of anaemia is rarely found (Piper *et al.*, 1987). Anaemia is probably only a factor with regard to fatigue when haemoglobin levels are extremely low (Bruera & MacDonald, 1988).

Day-time tiredness can be induced by *drugs* with a sedative (main or side) effect, including anti-emetics, analgesics or sleeping-agents (Aistars, 1987). Tiredness can also result from *insomnia*, which is a common problem of cancer patients (McCorkle & Quint, 1983; Cannici *et al.*, 1983; Kaye *et al.*, 1983; Silberfarb *et al.*, 1985). Among other things, difficulty getting to sleep and difficulty staying asleep might result from *pain*. A significant correlation ($r = .48$ $P < .0001$) has been found between the severity of pain and the intensity of fatigue (Blesch *et al.*, 1991). Finally, it was made plausible that *Immobilisation* was an important factor for a post-operative increase in heart rate during exercise which was significantly related to an increase in reports of fatigue (Christensen *et al.*, 1982; Zeiderman *et al.*, 1990).

To our knowledge, no one has yet addressed the issue of which somatic mechanisms might contribute to persistent fatigue after cancer-treatment, when no symptoms of disease can be found. Except for drug-use and insomnia, none of the somatic factors presented seems relevant for the explanation of this chronic fatigue.

When discussing the etiology of fatigue, many authors mention the possible influence of psychological factors. Depression in particular is considered to be a contributor to fatigue in cancer. Feelings of depression may result from the fact that one has a possibly fatal disease, and a depressed state of mind may induce fatigue. However, depression could not only be a cause, but also a result of persistent feelings of tiredness. Loss of function, loss of energy resulting directly from illness or its treatment may have this adverse psychological consequence. Finally, in cancer depression and fatigue may co-occur because both result from the same biological factors (Hayes, 1991).

As yet, no systematic studies have been performed to investigate the relationship between depression or anxiety and fatigue experienced by cancer patients. However, most study results suggest a relation between negative affect and fatigue. Preliminary results indicate that patients themselves notice a relation between psychological patterns and their fatigue experience (Piper, 1990). Women receiving treatment for breast cancer were asked what they believed contributed most directly to or caused their fatigue. Their answers indicated that changes in psychological patterns such as increased stress, worry, depression and anxiety contributed to fatigue most. Fatigue was found to be significantly related to mood (Cristensen, 1989; Blesch *et al.*, 1991). Nerenz *et al.* (1982) found tiredness to be strongly associated with the emotional distress experienced during chemotherapy treatment. Finally, patients whose energy level had not returned to normal after treatment for Hodgkin's disease, were also more likely to have elevated depression scores (Fobair *et al.*, 1986).

### Research issues

From the foregoing it can be concluded that research in the domain of fatigue in cancer is only in its infancy. Further research has to address issues such as (a) prevalence rate of fatigue at different stages of the disease, including follow-up data after successful treatment (b) the somatic, behavioral and psychological correlates of fatigue and (c) the consequences of this particular symptom for the well-being of the patient.

An important omission in most research on cancer fatigue is the lack of comparison-groups (Irvine *et al.*, 1991). To be able to determine the significance of the results obtained, the prevalence and intensity of fatigue in cancer patients need to be compared with the prevalence and intensity of fatigue in patients with other diseases or with non-patient populations. Prevalence rates between 14 and 34% of tiredness have been found in community surveys (Chen, 1986; Rillsdale, 1991). This means that the prevalence rates of fatigue in cancer patients should be rather high, to justify the conclusion that patients differ in their fatigue experience from the general population. Pickard *et al.* (1991) did compare the fatigue scores of women receiving chemotherapy for ovarian cancer, with the scores a convenience sample of apparently healthy adult women. The difference between the mean fatigue scores of both groups did not reach significance. This lack of difference may be explained satisfactorily by the small samples used in this study.

However, there is another possible explanation for the similarity in fatigue scores of patients and non-patients. When comparing the fatigue scores of cancer patients with those of other populations, or when comparing fatigue scores obtained before treatment with those obtained after treatment, an interpretation problem arises; the problem of a 'response shift' having occurred in the sample of cancer patients. The term 'response shift' refers to the change in a person's internal standard for determining his or her level of functioning on a given dimension (Breetvelt & van Dam, 1991). This shift in standard may result from a training course, an intervention or a major life-event. The experience of fatigue during radio- or chemotherapy, could change a patient's standard of measurement concerning fatigue. What has been perceived to be 'intense' fatigue before treatment, might be labelled 'slightly' fatigued after having experienced exhaustion during treatment. The possible occurrence of a 'response shift' complicates the interpretation of comparison data. These interpretation problems have been described in studies evaluating the effectiveness of training or therapy interventions. In this field of study methods have been recommended to ascertain whether a 'response shift' has occurred. Howard *et al.* (Breetvelt & Van Dam, 1991) advocated the use of 'then'-ratings, on which subjects indicated, in retrospect after the treatment, how they rated their functioning before treatment. As the 'then' pretreatment scores were assessed at the same time as the post-treatment scores, it is probable that these scores were assigned from the same perspective and therefore are free from 'response shift' bias. Further research has to indicate the applicability of techniques for the assessment of a 'response shift' with regard to symptom reporting.

### Concluding remarks

The hope is that good measurement and a better understanding of the course and correlates of fatigue may result in recommendations for interventions. These recommendations would then require systematic testing in populations of cancer patients. At this moment we can only speculate about possible beneficial interventions. Results have indicated that patients do no always expect fatigue to be a side-effect of treatment (Cassileth *et al.*, 1985; Love *et al.*, 1989; Tierney *et al.*, 1991). Preparatory information on what to expect in terms of fatigue during and after treatment, could enhance the ability of patients to cope with this symptom. Good information may also prevent unnecessary worries about fatigue indicating tumour growth or treatment failure.

In healthy individuals it is generally assumed that exercise is effective in reducing fatigue. So far, this assumption has been tested with cancer patients in preliminary studies only (Questad, 1983; McVicar & Winningham, 1984; Young & Sexton, 1991). Due to limitations of these studies no firm conclusions can be drawn. However, the results obtained are promising enough to stimulate further research.

The expectation as to the effect of physical exercise has been attributed a role in the maintenance of fatigue (Wessely *et al.*, 1990). Anticipating fatigue with certain activities, will lead to avoidance. Such an anticipation may be based on one or a few negative experiences, for instance after a too enthusiastic start of activities in the early phase of convalescence. Avoidance makes the person unfit, leading to easy development of fatigue when physical exercise is again undertaken.

Finally, studies evaluating the effectiveness of a psychotherapeutic intervention for cancer patients have found decreased fatigue-scores as a result of these interventions (Spiegel *et al.*, 1981; Forester *et al.*, 1985; Fawzy *et al.*, 1990). Therefore interventions aimed at reducing emotional distress or enhance coping responses may also be an effective way to decrease feelings of fatigue.

Further research might give information on those factors that differentiate between patients for whom the fatigue is in part the result of psychological or behavioral factors, from those patients whose fatigue is mainly somatic in origin. Being able to differentiate means that the interventions offered can be more customised to the needs of the individual patient.

At this moment no other conclusion remains then that our knowledge of fatigue as experienced by cancer patients is limited. Much research will be needed to clarify the undoubtedly complex somatic and psychological mechanisms responsible for the development, maintenance and treatment of fatigue.

### References

ACHARD, S. & ZITOUN, R. (1991). Main determinant factors accounting for quality of life or hematological patients during intensive care. *The 5th Conference of the European Society for Psychosocial Oncology*, Firenze.

AISTARS, J. (1987). Fatigue in cancer patients: A conceptual approach to a clinical problem. *Oncol. Nurs. Forum*, **14**, 25–30.

ANDERSEN, B.L. & TEWFIK, H.H. (1985). Psychological reactions to radiation therapy: reconsideration of the adaptive aspects of anxiety. *J. Personal. Soc. Psychol.*, **48**, 1024–1032.

APPELS, A. & MULDER, P. (1988). Excess fatigue as a precursor of myocardial infarction. *Eur. Heart. J.*, **9**, 758–764.

BERGLUND, G., BOLUND, C., FORNANDER, T., RUTQVIST, L.E. & SJÖDEN, P.-O. (1991). Late effects of adjuvant chemotherapy and postoperative radiotherapy on quality of life among breast cancer patients. *Eur. J. Cancer*, **27**, 1075–1081.

BLESCH, K., PAICE, J.A., WICKHAM, R., HARTE, N., SCHNOOR, D.K., PURL, S., REHWAIT, M., KOPP, P., MANSON, S., COVENY, S., MCHALE, M. & CAHILL, M. (1991). Correlates of fatigue in people with breast or lung cancer. *Oncol. Nurs. Forum*, **18**, 81–87.

BREETVELT, I.S. & VAN DAM, F.S.A.M. (1991). Underreporting by cancer patients: the case of response-shift. *Soc. Sci. Med.*, **32**, 981–987.

BREIJ, G.C.C. & DE VISSER-WOLF, P.J. (1990). *Investigation Hodgkin-Contactgroup*. Research Report, University of Utrecht.

BRUERA, E. & MACDONALD, R.N. (1988). Overwhelming fatigue in advanced cancer. *Am. J. Nursing*, **88**, 99–100.

CAMPELL, D.F., DIXON, J.K., SANDERFORD, L.D. & DENICOLA, M.A. (1984). Relaxation: Its effect on the nutrional status and performance status of clients with cancer. *J. Am. Diet. Ass.*, **84**, 201–204.

CANNICI, J., MALCOLM, R. & PEEK, L.A. (1983). Treatment of insomnia in cancer patients using muscle relaxation training. *J. Behavior Ther. Experim. Psychiatr.*, **14**, 251–256.

CASSILETH, B.R., LUSK, E.J., BODENHEIMER, B.J., FARBER, J.M., JOCHIMSEN, P. & MORRIN-TAYLOR, B. (1985). Chemotherapeutic toxicity – the relationship between patients' expectations and post-treatment results. *Am. J. Clin. Oncol.*, **8**, 419–425.

CHEN, M.K. (1986). The epidemiology of self-perceived fatigue among adults. *Preventive Med.*, **15**, 74–81.

CHIN, A., CRAANDIJK, M., FEESTRA, W.F.F. & LEER, J.W.H. (1990). De bestralingskater: prospectief onderzoek naar voorkomen en beloop. *Nederl. Tijdschr. Geneeskd.*, **134**, 1091–1094.

CHRISTENSEN, T., BENDIX, T. & KEHLET, H. (1982). Fatigue and cardiorespiratory function following abdominal surgery. *Br. J. Surg.*, **69**, 417–419.

DEVLEN, J., MAGUIRE, P., PHILIPS, P., CROWTHER, D. & CHAMBERS, H. (1987). Psychological problems associated with diagnosis and treatment of lymphomas. 1. retrospective; 2. prosepective. *Br. Med. J.*, **295**, 953–957.

FAWZY, F.I., COUSINS, N., FAWZY, N.W., KEMENY, M.E., ELASHOFF, R. & MORTON, D. (1990). A structured psychiatric intervention for cancer patients. *Arch. Gen. Psychiatry*, **47**, 720–725.

FOBAIR, P., HOPPE, R.T., BLOOM, J., COX, R., VAUGHESE, A. & SPIEGEL, D. (1986). Psychosocial problems among survivers of Hodgkin's disease. *J. Clin. Oncol.*, **4**, 805–814.

FORESTER, B., KORNFELD, D.S. & FLEISS, J.L. (1985). Psychotherapy during radiotherapy: Effects on emotional and physical distress. *Am. J. Psychiatr.*, **142**, 22–27.

FRIJDA, N.H. (1986). *The Emotions. Studies in Emotion and Social Interaction.* Cambridge University Press.

DE HAES, J.C.J.M., VAN KNIPPENBERG, F.C.E. & NEIJT, J.P. (1990). Measuring psychological and physical distress in cancer patients: structure and application of the Rotterdam Symptom Checklist. *Br. J. Cancer*, **62**, 1034–1038.

HAYES, J.R. (1991). Depression and chronic fatigue in cancer patients. *Primary Care*, **18**, 327–339.

HAYLOCK, P.F. & HART, L.K. (1979). Fatigue in patients receiving localized radiation. *Cancer Nursing*, 461–467.

IRVINE, D.M., VINCENT, L., BUBELA, N., THOMPSON, L. & GRAYDON, J. (1991). A critical appraisal of the research literature investigating fatigue in the individual with cancer. *Cancer Nursing*, **14**, 188–199.

JAMAR, S. (1989). Fatigue in women receiving chemotherapy for ovarian cancer. In Funk, S.G., Tornquist, E.M., Campagne, M.T., Archer Gopp, L. & Wiese, R.A. (eds). *Key Aspects of Comfort. Management of Pain Fatigue and Nausea.* New York: Springer Publishing Company.

KAYE, J., KAYE, K. & MADOW, L. (1983). Sleep patterns in patients with cancer and patients with cardiac disease. *J. Psychol.*, **114**, 107–113.

KING, K.B., NAIL, L.M., KREAMER, K., STROHL, R.A. & JOHNSON, J.E. (1985). Patients' description of the experience of receiving radiation therapy. *Onc. Nurs. Forum.*, **12**, 55–61.

KOBASHI-SCHOOT, J.A.M., HANEWALD, G.J.F.P., VAN DAM, F.S.A.M. & BRUNING, P.F. (1985). Assessment of malaise in cancer treated with radiotherapy. *Cancer Nursing*, **8**, 306–314.

KOGI, K., SAITO, Y. & MITSUHASHI, T. (1970). Validity of three components of subjective fatigue feelings. *J. Sci. Labour*, **46**, 251–270.

KRUPP, L.B., LAROCCA, N.G., MUIR-NASH, J. & STEINBERG, A.D. (1989). The fatigue severity scale. Application to patients with multiple sclerosis and systemic lupus erythematosus. *Arch. Neurol.*, **46**, 1121–1123.

LINSSEN, A.C.G., VAN DAM, F.S.A.M., ENGELSMAN, E., VAN BENTHEM, J. & HANEWALD, G.J.F.P. (1979). Leven met cytostatica. *Pharmaceut. Weekblad.*, **114**, 501–515.

LOVE, R.R., LEVENTHAL, H., EASTERLING, D.V. & NERENZ, D.R. (1989). Side effects and emotional distress during cancer chemotherapy. *Cancer*, **63**, 604–612.

MCCORKLE, R. & QUINT-BENOLIEL, J. (1983). Symptom distress, current concerns and mood disturbance after diagnosis of life threatening disease. *Soc. Sci. Med.*, **17**, 431–438.

MCVICAR, M. & WINNINGHAM, M. (1984). Effect of aerobic training on functional status of women with breast cancer. *Oncol. Nursing Forum Suppl.*, **11**,

MEYMAN, T.F. (1991). *About Fatigue*. Unpublished doctoral dissertation, University of Amsterdam.

MITCHELL, G.W. & GLICKSMAN, A.S. (1977). Cancer patients: knowledge and attitudes. *Cancer*, **40**, 61–66.

NAIL, L.M. & KING, K.B. (1987). Fatigue. *Seminars in Oncology Nursing*, **3**, 257–262.

NERENZ, D.R., LEVENTHAL, H. & LOVE, R.R. (1982). Factors contributing to emotional distress during cancer chemotherapy. *Cancer*, **50**, 1020–1027.

OBERST, M.T., HUGHES, S.H., CHANG, A.S. & MCCUBBIN, M.A. (1991). Self-care burden, stress appraisal and mood among persons receiving radiotherapy. *Cancer Nursing*, **14**, 71–78.

PEARSON, P.G. & BYARS, G.E. (1956). The development and validation of a checklist measuring subjective fatigue. (Report no. 56-115). School of aviation, USAF, Randolf AFB, Texas.

PECK, A. & BOLAND, J. (1977). Emotional reactions to radiation treatment. *Cancer*, **40**, 180–184.

PICKARD-HOLLEY, S. (1991). Fatigue in cancer patients. A descriptive study. *Cancer Nursing*, **14**, 13–19.

PIPER, B.F. (1990). Fatigue. Trans-cultural implications for nursing interventions. *Presentation at the Sixth International Conference on Cancer Nursing*. Amsterdam.

PIPER, B.F., LINDSEY, A.M., DODD, M.J., FERKETICH, S., PAUL, S.M. & WELLER, S. (1989). The development of an instrument to measure the subjective dimension of fatigue. In Funk, S.G., Tornquist, E.M., Campagne, M.T., Archer Gopp, L. & Wiese, R.A. (eds). *Key Aspects of Comfort. Management of Pain Fatigue and Nausea*. New York: Springer Publishing Company.

PIPER, B.F., LINDSEY, A.M. & DODD, M.J. (1987). Fatigue mechanisms in cancer patients: Developing nursing theory. *Oncol. Nursing Forum*, **14**, 17–23.

PIPER, B.F. (1988). Fatigue in cancer patients: current perspective on measurement and management. Fifth National Conference of Cancer Nursing. *Monograph on Nursing Management of Common Problems: State of the Arts*. New York: American Cancer Society.

QUESTAD, K.A. (1983). An empirical study of a rehabilitation program for fatigue related to cancer. *Dissertation Abstract International*, **44**, 1974–1975.

RHODES, V.A., WATSON, P.M. & HANSON, B.M. (1988). Patients' descriptions of the influence of tiredness and weakness on self-care abilities. *Cancer Nursing*, **11**, 186–194.

RHOTEN, D. (1982). Fatigue and the postsurgical patient. In Norris, C.M. (ed.) *Concept Clarification in Nursing*. Rockville MD, Aspen Publishers Inc, 277–300.

RILLSDALE, L. (1991). Tired all the time. *Br. Med. J.*, **303**, 1490–1491.

SILBERFARB, P.M., HAURI, P.J., OXMAN, T.E. & LASH, S. (1985). Insomnia in cancer patients. *Soc. Sci. Med.*, **20**, 849–850.

SPIEGEL, D., BLOOM, J.R. & YALOM, I. (1981). Group support for patients with metastatic cancer. A randomized prospective outcome study. *Arch. Gen. Psychiatr.*, **38**, 527–533.

SRIVASTAVA, RANI HAJELA. (1989). Fatigue in end-stage renal disease patients. In Funk, S.G., Tornquist, E.M., Campagne, M.T., Archer Gopp, L. & Wiese, R.A. (eds). *Key Aspects of Comfort. Management of Pain, Fatigue and Nausea*. New York: Springer Publishing Company.

TIERNEY, A.J., LEONARD, R.C.F., TAYLOR, J., CLOSS, S.J., CHETTY, U. & RODGER, A. (1991). Side effects expected and experiences by women receiving chemotherapy for breast cancer. *Br. Med. J.*, **302**, 151–152.

WESSELY, S., BUTLER, S., CHALDER, T. & DAVID, A. (1990). The congnitive behavioral management of the post viral fatigue syndrome. In Jenkins, R. & Mowbray, J. (eds). *The Postviral Fatigue Syndrome*. Chichester: Wiley.

YASKO, Y.M. & GREENE, P. (1987). Coping with problems related to cancer and cancer treatment. *CA-A J. Clin.*, **37**, 106–125.

YOUNG-MCAUGHAN, S. & SEXTON, D.L. (1991). A retrospective investigation of the relationship between aerobic exercise and quality of life in women with breast cancer. *Oncol. Nursing Forum*, **18**, 751–757.

ZEIDERMAN, M.R., WELCHEW, E.A. & CLARK, R.G. (1990). Changes in cardiorespiratory and muscle function associated with the development of postoperative fatigue. *Br. J. Surgery*, **77**, 576–580.